# Effects of Whey Protein, Leucine, and Vitamin D Supplementation in Patients with Sarcopenia: A Systematic Review and Meta-Analysis

**DOI:** 10.3390/nu15030521

**Published:** 2023-01-19

**Authors:** Min Cheol Chang, Yoo Jin Choo

**Affiliations:** Department of Physical Medicine and Rehabilitation, College of Medicine, Yeungnam University, Daegu 42415, Republic of Korea

**Keywords:** sarcopenia, whey protein, leucine, vitamin D, meta-analysis

## Abstract

(1) Background: In this study, a meta-analysis was performed to investigate the effects of whey protein, leucine, and vitamin D in sarcopenia; (2) Methods: We searched PubMed, Cochrane Library, Embase, and Scopus databases and retrieved studies published until 5 December 2022. Randomized controlled trials were included to evaluate muscle mass, strength, and function, after using whey protein, leucine, and vitamin D supplementation in patients with sarcopenia; (3) Results: A total of three studies including 637 patients reported the effectiveness of using whey protein, leucine, and vitamin D supplementation in patients with sarcopenia. Without considering whether or not a physical exercise program was combined with nutritional supplementation, no significant differences in grip strength or short physical performance battery (SPPB) scores between the experimental and control groups were noted. However, appendicular muscle mass significantly improved in the experimental group compared to the control group. The results were analyzed according to the presence or absence of a concomitant physical exercise program. With the use of a concomitant physical exercise program, handgrip strength and SPPB scores in the experimental group significantly improved when compared to the control group. In contrast, when physical exercise was not combined, there was no significant improvement in the handgrip strength and SPPB scores of patients with sarcopenia. In addition, the appendicular muscle mass significantly increased regardless of the presence of a concomitant physical exercise program; (4) Conclusions: Whey protein, leucine, and vitamin D supplementation can increase appendicular muscle mass in patients with sarcopenia. In addition, combining a physical exercise program with whey protein, leucine, and vitamin D supplementation can improve muscle strength and function.

## 1. Introduction

Sarcopenia is an age-associated syndrome characterized by a progressive and generalized loss of skeletal muscle mass and strength [1,2]. Sarcopenia results in an impaired ability to perform activities of daily living, an increased risk of disability, loss of independence, and a decreased quality of life [1,2]. The etiology of sarcopenia is multifactorial, including hormonal changes, neurological decline, a mitochondrial decline in skeletal muscles, fatty infiltration, a decline in activity, chronic illnesses, and poor nutrition [3]. Aerobic exercise and resistance training are known to increase muscle mass and enhance physical function [4,5]. Aerobic exercise improves aerobic capacity by inducing adenosine triphosphate production in the mitochondria of skeletal muscle, inhibits their apoptotic pathways, and contributes to increasing the expression of autophagic proteins [5]. These molecular factors suggest that aerobic exercise has a protective effect against sarcopenia [5]. Resistance exercise is an exercise wherein muscles must maintain contraction or work against an applied force or weight [4]. Compared to resistance exercise, aerobic exercise has less effect on muscle strength or mass, but resistance exercise has a higher risk of injury and lower participation rates than aerobic exercise [5]. In addition, resistance exercise may be less effective in the elderly due to the lack of mammalian targets of rapamycin signaling involved in muscle protein synthesis during resistance exercise. Therefore, when exercising for the treatment of sarcopenia, aerobic exercise and resistance exercise should be appropriately combined [5]. Moreover, previous studies have reported that oral nutritional interventions have the potential to decrease functional disability and improve body composition [6,7,8]. In 2020, Ellis et al. [6] randomly assigned 31 people aged 65 years or older to take an amino acid supplement twice daily for six months or a placebo. The group taking the amino acid supplement showed significant improvement in physical function compared to the group taking the placebo. In addition, only the group taking amino acid supplements showed an increase in arm lean mass. In 2013, Kim et al. [7] investigated the effects of nutritional supplements on 87 people aged over 65 years or older who met frailty criteria (usual gait speed, <0.6 m/s; mini nutritional assessment, <24). Participants were randomly assigned to either a nutritional supplement group containing protein and essential amino acids or a control group that received no treatment. The nutritional supplement was consumed twice daily for 12 weeks, and the physical function of the supplement intake group improved by 5.9%, but no change was observed in the control group. In addition, gait speed and activity performance speed increased in the supplement intake group but deteriorated in the control group. In 2017, Ottestad et al. [8] evaluated the effect of protein-enriched milk on 36 people aged 70 years or older with reduced physical strength or activity capacity. Participants were randomly assigned to either a group to consume protein-enriched milk or a control group to drink a carbohydrate beverage. Both protein and carbohydrate drinks were consumed twice daily for 12 weeks, and both the supplement intake group and the control group showed improvements in body composition and muscle strength evaluations.

A nutritional recommendation for the elderly is to increase their daily protein intake (1–1.2 g/kg/day) with large amounts of essential amino acids such as leucine [9]. Leucine is an independent and potent modulator of protein turnover, particularly protein anabolism [10,11]. It is suggested that a maximum of 2.8–3 g of leucine should be provided at least twice daily [10,11]. A high-quality oral nutritional supplementation (ONS) can be considered for an adequate intake of high-quality protein or amino acids in the elderly [12]. In particular, patients with sarcopenia frequently have an inadequate food intake; therefore, ONS can be particularly helpful for them to ensure that they have a sufficient intake of protein and amino acids [13]. Whey protein is a valuable protein source with greater anabolic properties due to its higher essential amino acid content and faster digestion than other protein sources [14]. In addition, leucine is highly expressed in whey proteins.

In addition, vitamin D supplementation (at least 800–1000 IU/day) should be considered in older adults [15]. Vitamin D synergizes with leucine by enhancing protein anabolism [15]. Vitamin D can also potentially improve physical activity and muscle function in the elderly [16]. Thus, it was suggested that combining whey protein, leucine, and vitamin D supplementation would be beneficial to enhance physical function and increase muscle mass. However, the previous studies on this topic have reported conflicting results [13].

In this study, we performed a systematic review and meta-analysis to evaluate the effectiveness of whey protein, leucine, and vitamin D supplementation in patients with sarcopenia.

## 2. Materials and Methods

### 2.1. Search Strategy

This systematic review and meta-analysis conformed to the recommendations of the Preferred Reporting Items for Systematic Review and Meta-analysis (PRISMA) [17]. The protocol was registered on the international platform of registered systematic reviews and meta-analysis protocols (registration number: INPLASTY2022120016). In this study, the PICO (population, intervention, comparison, outcome) model for developing the search strategy was set as follows: (1) “population” referred to patients diagnosed with sarcopenia; (2) “intervention” referred to whey protein, leucine, and vitamin D supplementation; (3) “comparison” referred to isocaloric supplementation; and (4) “outcome” referred to changes in muscle mass, muscle strength, and muscle function. This systematic review and meta-analysis were conducted in accordance with the Preferred Reporting Items for Systematic Reviews and Meta-analysis (PRISMA) guidelines. We searched PubMed, Cochrane Library, Embase, and Scopus databases for studies published up to 5 December 2022. The search terms were as follows: “whey protein”, “leucine”, “vitamin D”, “nutrition”, “sarcopenia”, “muscle strength”, “muscle function”, and “muscle mass” (Supplementary 1).

### 2.2. Inclusion and Exclusion Criteria

Studies that satisfied all of the following selection criteria were included in this meta-analysis: (1) randomized controlled trials involving patients with sarcopenia; (2) studies comparing the effectiveness of whey protein, leucine, and vitamin D supplementation with isocaloric supplementation; (3) studies evaluating muscle mass, muscle strength, or muscle function after the intervention; (4) studies with complete text; and (5) studies written in English. Studies published as case reports, reviews, conference presentation, letters, or other undistinctive forms and studies reporting insufficient data or results were excluded.

### 2.3. Data Extraction

All search results were exported to the EndNote 20 software tool (Clarivate, London, United Kingdom). After excluding duplicate articles using the deduplication function of EndNote 20, two reviewers (M.C.C. and Y.J.C.) independently evaluated potentially eligible studies that met the inclusion criteria. Studies were selected after primarily reviewing titles and abstracts. Thereafter, eligibility was confirmed through a full-text review of the selected studies. In case of disagreement, the decision was made by consensus between the two reviewers (M.C.C. and Y.J.C). An increased muscle mass in sarcopenia was identified as an excellent prognostic indicator for patients with sarcopenia, and appendicular muscle mass was investigated. Grip strength and short physical performance battery (SPPB) scores were assessed to determine the degree of improvement in muscle strength and function. In cases of insufficient data reported from an included study, we attempted to contact the authors.

### 2.4. Quality and Level of Evidence Assessments

The methodological quality of selected studies was evaluated using the criteria described in the Cochrane Handbook for Systematic Reviews of Interventions to assess potential bias [18]. The domains to evaluate the risk of bias were as follow: (1) random sequence generation and allocation concealment (selection bias), (2) blinding of participants and personnel (performance bias), (3) blinding of outcome assessment (detection bias), (4) incomplete outcome data (attrition bias), (5) selective reporting (reporting bias), and (6) other bias. Two independent reviewers performed these evaluations (M.C.C. and Y.J.C.), and discrepancies were resolved through discussion.

### 2.5. Analyses

Review management software (RevMan 5.3, The Cochrane Collaboration, Copenhagen, Denmark) was used for the statistical analysis of the pooled data. Sensitivity analysis was performed by identifying studies and removing them one by one. For each analysis, a heterogeneity test was performed using *I*^2^ statistics, which measured the extent of inconsistencies among the results. When the *I*^2^ values were ≤50%, the pooled data were considered homogeneous, and a fixed-effects model was adopted. In contrast, when *I*^2^ values were more than 50%, the pooled data were considered to have substantial heterogeneity, and the random-effects model was applied for the data analyses. As there were both studies that included and did not include exercise interventions, a subgroup analysis was performed to analyze the heterogeneity between these studies. The analyzed data included continuous variables. Therefore, the resultant data were presented as standard mean differences (SMDs) and 95% confidence intervals (Cis). Statistical significance was set at *p*-value < 0.05. All meta-analysis results were visualized as a forest plot.

A funnel plot and Egger’s test were used to evaluate publication bias using R software, version 4.1.2 (R Foundation for Statistical Computing, Vienna, Austria). A funnel plot was used to determine the publication bias of individual studies based on the pooled estimates. Egger’s test, which statistically assessed the symmetry of the funnel plots, could only be performed when three or more studies were included in the meta-analysis. A *p*-value of <0.05 in an Egger’s test indicates potential publication bias.

## 3. Results

### 3.1. Study Selection

**Figure 1:**
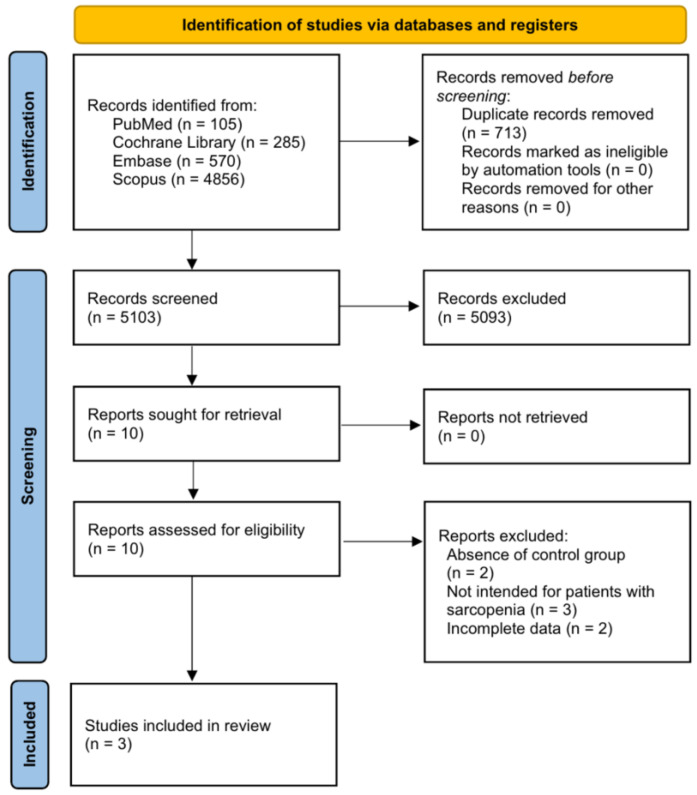
Flow diagram showing the search results of the meta-analysis.

From 5816 studies retrieved using the abovementioned keywords, 5103 studies were selected after excluding duplicate articles. After confirming the title and abstract, 5093 articles were excluded, and two more articles without a control group were excluded from the remaining ten studies. In addition, three studies that did not include patients with sarcopenia and two studies that could not be included in the meta-analysis owing to insufficient data were excluded. Therefore, three papers [19,20,21] were included in this study (Figure 1).

### 3.2. Study Description

In this meta-analysis, a group of patients who received whey protein, leucine, and vitamin D supplementation was defined as the experimental group, and a group of patients who received an isocaloric product was defined as the control group.

In 2015, Bauer et al. [19] conducted a 13-week study of 380 elderly sarcopenic participants with mobility limitations. The mean age of all participants was 77.7 years, the mean body mass index (BMI) was 26.1 kg/m^2^, and the mean mini mental state examination (MMSE) score was 29, with no abnormalities in cognitive function. Participants were randomly assigned to an experimental group (*n* = 184) who received a vitamin D and leucine-enriched whey protein nutritional supplementation and a control group (*n* = 196) who received an isocaloric product. Per serving, the experimental group received 20.7 g of whey protein, 2.8 g of total leucine, 800 IU of vitamin D, 10.6 g of essential amino acids, 9.4 g of carbohydrates, 3 g of fat, 1.3 g of fiber, 1.3 g of minerals, and 0.3 g of trace elements. The isocaloric product provided to the control group contained 31.4 g of carbohydrates, 3 g of fat, and 0.7 g of some minerals, and no whey protein, leucine, vitamins, essential amino acids, fiber, or trace elements. Both products were provided as 40 g powder with 150 kcal, and the participants consumed the products before every breakfast and lunch. There was no physical exercise intervention other than nutritional supplementation.

In 2016, Rondanelli et al. [20] conducted a 12-week study of 130 elderly patients with sarcopenia. The mean age of all participants was 80.3 years, and the mean BMI was 23.89 kg/m^2^. The mean MMSE score was 21.1, and participants with mild or no cognitive impairment were included. Participants were randomly assigned and included 69 in the experimental group and 61 in the control group. The nutritional supplementation provided to the experimental group was 112 kcal per 32 g and that consisted of 22 g of whey protein, essential amino acids including 4 g of leucine, 100 IU of vitamin D, 4.7 g of carbohydrates, 0.4 g of fat, 2.2 g of fiber, and 0.4 g of minerals. The control group received an isocaloric product consisting of maltodextrin that had the same taste and appearance as the nutritional supplementation received by the experimental group. Participants received either a nutritional supplementation or a placebo administered orally once daily at mealtime. In addition, a moderate intensity of physical fitness and muscle mass enhancement training program was provided to all participants. The exercise was conducted 5 times a week for 12 weeks, and consisted of 5 min of warm-up, 5 min of strengthening exercise, 5 min of balance and gait training, and 5 min of cool-down. Exercises such as toe and heel raising and knee and hip flexion were performed while sitting on a chair or standing behind the chair and holding the back of the chair. In order to develop lower extremity strength, ankle weight exercises with fixed weights applied to the ankles were also included. In addition, resistance bands were used to flex or extend joints to strengthen the upper and lower extremity, and balance and gait training, including one-legged standing or tandem stands, were also conducted.

In a study conducted by Rondanelli et al. in 2020, [21] 127 elderly patients with sarcopenia participated. The mean age of the participants was 81.5 years, the mean BMI was 21.6 kg/m^2^, and the mean MMSE score was 21.9, indicating no severe cognitive impairment. Sixty-four patients were randomly assigned to the experimental group and sixty-three to the control group. Participants received physical exercise interventions along with whey protein-based nutritional supplementation rich in leucine and vitamin D, or isocaloric product, with interventions lasting at least 4 weeks and up to 8 weeks. The nutritional supplementation provided to the experimental group included 20 g of whey protein, 2.8 g of leucine, 800 IU of vitamin D, 9 g of carbohydrate, 3 g of fat, and a mixture of minerals. The placebo given to the control group was an isocaloric product containing maltodextrin. The nutritional supplementation and placebo both consisted of 40 g of powder, 150 kcal of energy, and were administered in the morning and afternoon. Physical exercise consisted of an individualized moderate-level physical fitness and muscle mass enhancement training program. Physical exercise was conducted in 20 min sessions five times a week. After warming up for 5 min, toe raises, heel raises, knee raises, knee extension, hip flexion and lateral leg raises, knee flexion and extension with a weight of 0.5 to 1.5 kg on the ankle, upper and lower extremity flexion and extension exercises using resistance bands were performed for 5 to 10 min. Afterward, balance and gait training, such as standing on one leg or moving weight in multiple directions, was performed for 5 to 10 min, followed by a 5 min cool-down.

The detail characteristics of the selected studies are presented in Table 1.

### 3.3. Risk of Bias

**Figure 2:**
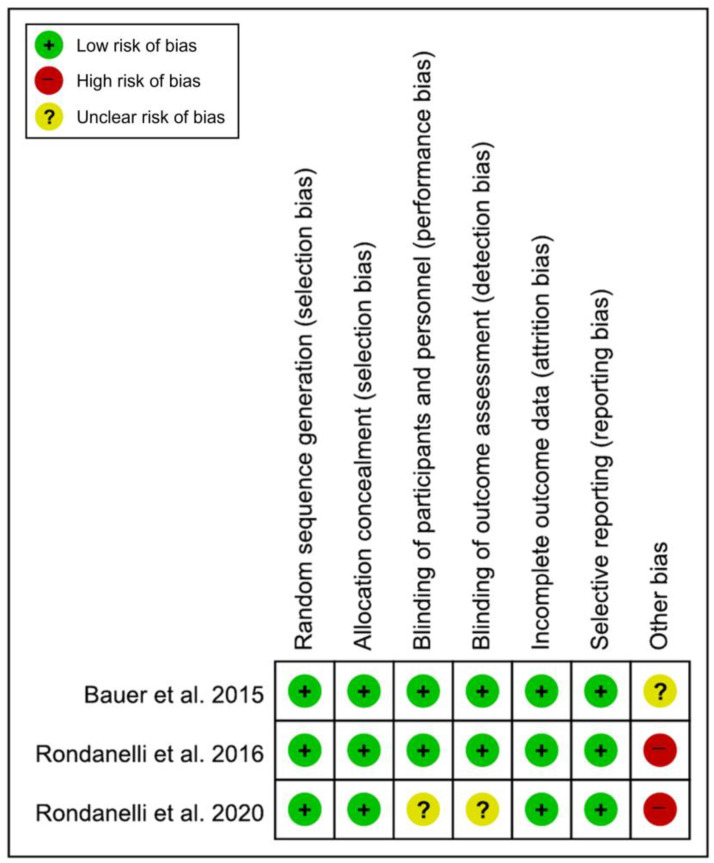
Results of the quality assessment of the included studies [19,20,21].

All included studies [19,20,21] had a low risk of bias in the domains of random sequence generation, allocation concealment, incomplete outcome data, and selective reporting. In the domains of the blinding of participants and personnel and the blinding of outcome assessments, two studies [19,20] had a low risk of bias, while another study [21] had an unclear risk of bias. There was no low risk of bias in the domains of other bias; two studies [20,21] had a high risk of bias, and the other study [19] had an unclear risk of bias (Figure 2). Of the 21 domains across all studies, 16 domains (76.2%) were determined to be low risk. Therefore, the overall risk of bias was determined to be low, and the studies included in this meta-analysis were assessed as high quality.

### 3.4. Meta-Analysis Results

**Figure 3:**
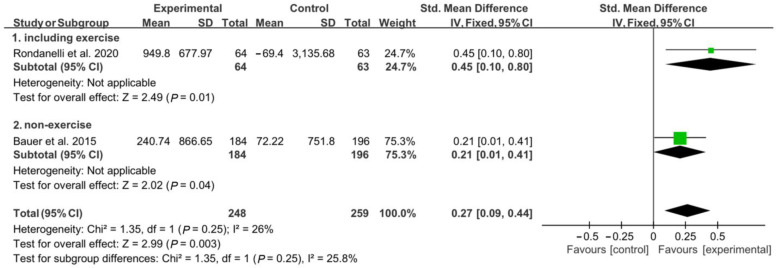
Forest plot showing the results of appendicular muscle mass after use of whey protein, leucine, and vitamin D in patients with sarcopenia [19,21]. Numbers in bold indicate analysis results.

In the analysis of appendicular muscle mass, 127 patients (64 in the experimental group and 63 in the control group) participated in a concomitant physical exercise program using whey protein, leucine, and vitamin D supplementation. In total, 380 patients (184 patients from the experimental group and 196 from the control group) received only nutritional supplementation. When analyzed without considering the involvement of a physical exercise program combined with nutritional supplementation, there was a significant difference between the experimental group and the control group regarding changes in appendicular muscle mass [SMD, 0.27; 95% CI, 0.09 to 0.44; *p* = 0.003; *I*^2^ = 26%]. Subgroup analysis was conducted, and the combination of nutritional supplementation and physical exercise programs was considered. Significant improvements were noted in the experimental group compared to the control group; these improvements occurred both with and without the combination of the physical exercise program [SMD, 0.45; 95% CI, 0.10 to 0.80; *p* = 0.01; *I*^2^ = N/A; and SMD, 0.21; 95% CI 0.01 to 0.41; *p* = 0.04; *I*^2^ = N/A, respectively] (Figure 3).

**Figure 4:**
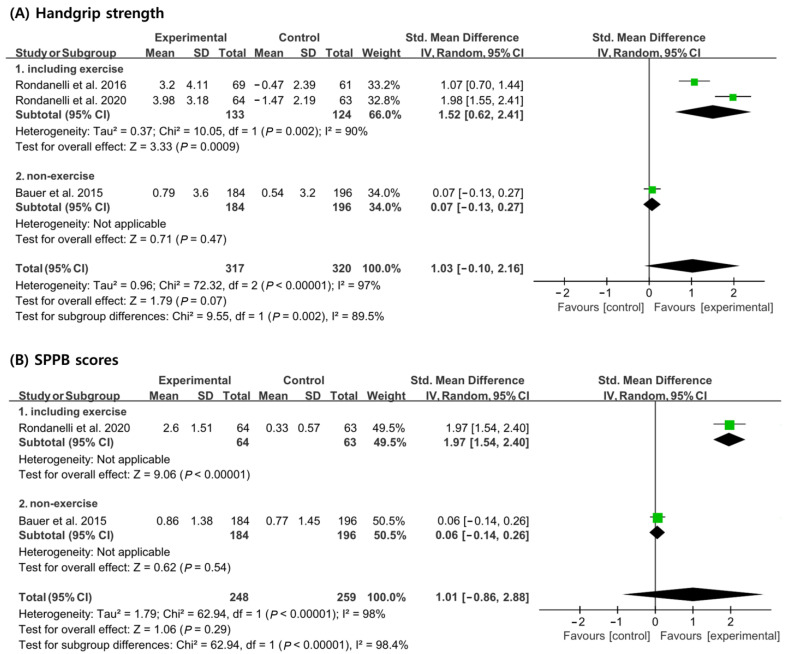
Forest plot showing the results of (**A**) handgrip strength [19,20,21] and (**B**) SPPB scores [19,21] after use of whey protein, leucine, and vitamin D in patients with sarcopenia. Numbers in bold indicate analysis results.

In the analysis of handgrip strength, 257 patients (133 patients from the experimental group and 124 patients from the control group) received a concomitant physical exercise program using whey protein, leucine, and vitamin D supplementation. In total, 380 patients (184 patients from the experimental group and 196 patients from the control group) did not receive a concomitant physical exercise program. When analyzed without considering nutritional supplementation combined with exercise, no significant differences in handgrip strength improvement were noted between the experimental and the control groups [SMD, 1.03; 95% CI, −0.10 to 2.16; *p* = 0.07; *I*^2^ = 97%]. However, when a physical exercise program was used in combination with nutritional supplementation, the experimental group showed significant improvements in handgrip strength compared to the control group [SMD, 1.52; 95% CI, 0.62 to 2.41; *p* = 0.0009; *I*^2^ = 90], but there was no significant difference between the experimental group and the control group when the physical exercise program was not combined [SMD, 0.07; 95% CI, −0.13 to 0.27; *p* = 0.47; *I*^2^ = N/A] (Figure 4A).

In the analysis of SPPB scores, a total of 127 patients (64 patients from the experimental group and 63 patients from the control group) received a concomitant physical exercise program using whey protein, leucine, and vitamin D supplementation. In total, 380 patients (184 patients from the experimental group and 196 patients from the control group) did not receive a concomitant physical exercise program. When analyzed without considering if a physical exercise program was performed in conjunction with nutritional supplementation, no significant difference in SPPB scores between the experimental group and the control group [SMD, 1.01; 95% CI, −0.86 to 2.88; *p* = 0.29; *I*^2^ = 98] were noted. However, when a physical exercise program was performed with nutritional supplementation, the SPPB scores showed significant improvements in the control group compared to the experimental group [SMD, 1.97; 95% CI, 1.54 to 2.40; *p* < 0.00001; *I*^2^ = 90]. However, the SPPB scores showed no significant improvement between the control and experimental groups when only nutritional supplementation was administered [SMD, 0.06; 95% CI, −0.14, 0.26; *p* = 0.54; *I*^2^ = N/A] (Figure 4B).

### 3.5. Publication Bias

**Figure 5:**
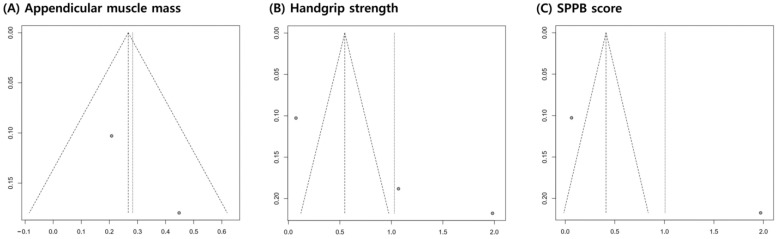
Graphic funnel plot of the included studies. The x-axis represents the standardized mean difference, and the y-axis represents the standard error.

The funnel plots for appendicular muscle mass, handgrip strength, and SPPB scores were symmetrical (Figure 5). In addition, the *p*-value of Egger’s test for grip strength was 0.1274, indicating insignificant publication bias.

## 4. Discussion

In the current meta-analysis, without considering whether a physical exercise program was performed combined with nutritional supplementation, whey protein, leucine, and vitamin D supplementation could effectively increase appendicular muscle mass in patients with sarcopenia. However, this did not significantly improve the handgrip strength or physical performance. An analysis of the results according to the presence of a concomitant physical exercise program, with a concomitant physical exercise program, indicated that the consumption of whey protein, leucine, and vitamin D supplementation significantly improved handgrip strength and physical performance in patients with sarcopenia. In contrast, when physical exercise was not combined with whey protein, leucine, or vitamin D supplementation, its consumption did not significantly improve the handgrip strength and physical performance in patients with sarcopenia. Appendicular muscle mass significantly increased, regardless of a concomitant physical exercise program.

Sufficient protein intake is crucial for retaining muscle mass and function in older patients [22,23,24]. The intake of whey protein and leucine reportedly provides sufficient quantities of essential amino acids, especially leucine, which is essential for appropriate muscle synthesis [25,26,27]. Additionally, maintaining optimal levels of serum 25-hydroxyvitamin D concentrations (50–75 nmol/L) is necessary to maintain the adequate strength of extremity muscles and enhance protein anabolism [28]. Accordingly, we believe that using whey protein, leucine, and vitamin D supplementation can help preserve muscle mass and improve muscle function in sarcopenic patients. 

According to our results, combining whey protein, leucine, and vitamin D supplementation with physical exercise in sarcopenia patients enhances muscle power and physical function. Furthermore, the appendicular muscle index increased significantly in the presence of concomitant physical exercise in patients with sarcopenia (with concomitant physical exercise, SMD = 0.45; without concomitant physical exercise, SMD = 0.21). Therefore, in our opinion, in addition to the consumption of whey protein, leucine, and vitamin D supplementation, physical exercise should be combined in sarcopenic patients. The consumption of whey protein, leucine, and vitamin D supplementation alone, without concomitant physical exercise, is limited in its ability to induce a positive therapeutic effect in sarcopenic patients.

In conclusion, consuming whey protein, leucine, and vitamin D supplements can increase appendicular muscle mass in sarcopenic patients. Furthermore, combining physical exercise with whey protein, leucine, and vitamin D supplementation significantly improves muscle power and performance. Therefore, clinicians should consider recommending whey protein, leucine, and vitamin D supplementation with physical exercise to their sarcopenic patients. Our study is the first meta-analysis to investigate the effects of whey protein, leucine, and vitamin D supplementation in patients with sarcopenia. However, our study is limited in that only a small number of previous studies were included. Therefore, further clinical studies should be conducted to confirm the effects of whey protein, leucine, and vitamin D supplementation in patients with sarcopenia.

## Figures and Tables

**Table 1:** Characteristics of selected studies.

Study	Study Design	Participant (Mean Age, M:F)	Intervention	Follow-Up Period	Measured Outcomes
Bauer et al., 2015 [19]	RCT	E: 77.3 ± 6.7 y, 64:120 C: 78.1 ± 7.0 y, 67:129	E: 20.7 g of whey protein, 2.8 g of total leucine, 800 IU of vitamin D, 10.6 g of essential amino acid, 9.4 g of carbohydrates, 3 g of fat, a mixture of vitamins, 1.3 g of fibers, 1.3 g of minerals, and 0.3 g of trace elements C: isocaloric product with 31.4 g of carbohydrates, 3 g of fat, and 0.7 g of some minerals	0, 7, 13 weeks	Hand grip strength, SPPB score, balance test, gait speed, change in appendicular muscle mass, serum 25-hydroxyvitamin D, serum IGF-1, non-supplementary dietary energy intake, non-supplementary dietary protein intake, total dietary energy intake including supplement, total dietary protein intake including supplement
Rondanelli et al., 2016 [20]	RCT	E: 80.77 ± 6.29 y, 29:40 C: 80.21 ± 8.54 y, 24:37	E: 22 g of whey protein, 4 g of leucine, 100 IU of vitamin D, 4.7 g of carbohydrates, 0.4 g of fat, 2.2 g of fibers, and 0.4 g of minerals C: isocaloric product with maltodextrin	0, 12 weeks	Handgrip strength, fat-free mass, fat mass, body weight, body mass index, waist circumference, relative skeletal muscle mass, activities of the daily living score, mini nutritional assessment, energy intake, protein intake, *C*-reactive protein, insulin-like growth factor I, fat intake, carbohydrates intake, vitamin D intake, SF-36
Rondanelli et al., 2020 [21]	RCT	E: 81.0 ± 7.0 y, 26:38 C: 82.0 ± 5.0 y, 17:46	E: 20 g of whey proteins, 2.8 g of leucine, 800 IU of vitamin D, 9 g of carbohydrates, 3 g of fat, a mixture of minerals C: isocaloric product with maltodextrin	Upon admission and discharge (for at least 4 weeks and up to 8 weeks)	Handgrip strength, SPPB score, Barthel index, chair stand test, timed up and go test, Tinetti scale, body weight, mini-mental state examination, trail-making test, activities of the daily living score, appendicular muscle mass, skeletal muscle mass index, mini nutritional assessment, energy intake, protein intake, *C*-reactive protein, 25-hydroxyvitamin D, total cholesterol, albumin, creatinine, duration of rehabilitation, length of stay, SF-12

M, male; F, female; E, experimental group; C, control group; y, years; SPPB, short physical performance battery; SF, short-form general health survey.

## Data Availability

All the data are available in the manuscript.

## Supplementary Materials

The following supporting information can be downloaded at: https://www.mdpi.com/article/10.3390/nu15030521/s1, Supplementary 1: Search strategy.
