# Supplementary material for: Effects of Whey Protein, Leucine, and Vitamin D Supplementation in Patients with Sarcopenia: A Systematic Review and Meta-Analysis
Source: Nutrients. 2023 Jan 19;15(3):521. doi: 10.3390/nu15030521 (PMC9920795; doi:10.3390/nu15030521)
Supplement: Supplementary file 1 [file nutrients-15-00521-s001.zip › nutrients-2129980-supplementary.pdf]

Supplementary 1. Search strategy

A search strategy was developed to identify studies that reported the effectiveness of whey protein, leucine, and vitamin D supplementation in patients with sarcopenia.

The search keywords were combined as follows:

〞whey protein〞 OR 〞leucine〞 OR 〞vitamin D〞

AND

〞sarcopenia〞

AND

〞muscle mass〞 OR 〞muscle strength〞 OR 〞muscle function〞

The search keywords were devised using a combination of subject indexing terms in the titles and abstracts. For the search terms were referenced [A Castro LH, S de Araújo FH, M Olimpio MY, et al. Comparative Meta-Analysis of the Effect of Concentrated, Hydrolyzed, and Isolated Whey Protein Supplementation on Body Composition of Physical Activity Practitioners. Nutrients. 2019;11:2047. "Table S1. Details of the central search strategy adapted to the databases"], [Guo Y, Fu X, Hu Q, et al. The Effect of Leucine Supplementation on Sarcopenia-Related Measures in Older Adults: A Systematic Review and Meta-Analysis of 17 Randomized Controlled Trials. Front Nutr. 2022;9:929891. "Supplementary table 1. Literature search strategy for meta-analysis"], [Cheng SH, Chen KH, Chen C, et al. The Optimal

Strategy of Vitamin D for Sarcopenia: A Network Meta-Analysis of Randomized Controlled Trials. Nutrients. 2021;13(10):3589. “Supplementary text 1. Search strategy”], and [Wang H, Huang WY, Zhao Y. Efficacy of Exercise on Muscle Function and Physical Performance in Older Adults with Sarcopenia: An Updated Systematic Review and Meta-Analysis. Int J Environ Res Public Health. 2022;19(13):8212. “Supplementary S1. Search strategy”].

Search strategy for: A Castro LH, S de Araújo FH, M Olimpio MY, et al. Comparative Meta-Analysis of the Effect of Concentrated, Hydrolyzed, and Isolated Whey Protein Supplementation on Body Composition of Physical Activity Practitioners. Nutrients. 2019;11:2047. “Table S1. Details of the central search strategy adapted to the databases”

1) PubMed<br><br>(active filters: “clinical trial” and “humans”) / (active options: “include references”). Date of execution: 2018/12/10.<br><br>#Strategy<br><br>((((“physic* activity” OR “physic* exercise” OR training OR exercise OR coaching OR “resistance training” OR “resistance exercise*” OR gym* OR fit* OR crossfit OR “weight lifting” OR “nonsedentary” OR athlete)) AND (“whey protein” OR whey OR “protein supplement*” OR “whey supplement*” OR casein OR “casein supplement*” OR “whey intake” OR “casein intake” OR “protein intake” OR “whey concentrate” OR “casein concentrate” OR “concentrate protein” OR “whey isolated” OR “casein isolated” OR “isolated protein” OR “hydrolyzed whey” OR “hydrolyzed casein” OR “hydrolyzed protein” OR milk OR “milk protein” OR soy OR “soy protein”)) AND

(muscle* OR “mass gain” OR “muscle* gain” OR “muscular gain” OR “muscle* strength*” OR “muscular* strength*” OR “hypertrophy” OR “lean mass” OR “body composition” OR “lean body mass” OR “lean body tissue” OR “fat-free mass” OR “fat free mass” OR “body weight” OR “body mass” OR “skeletal muscle*”)) NOT (child*[Title] OR pediatric*[Title] OR elder*[Title] OR aged[Title] OR rat*[Title] OR mice*[Title])

2) COCHRANE Trials

(active options: “search for similar words” and “include references”). Date of execution: 2018/12/10.

#Strategy

(“physical activity” OR “physical exercise” OR training OR exercise OR coaching OR “resistance training” OR “resistance exercise” OR gym OR fit OR fitness OR crossfit OR “weight lifting” OR “non-sedentary” OR athlete) AND (“whey protein” OR whey OR “protein supplement” OR “whey supplement” OR casein OR “casein supplement” OR “whey intake” OR “casein intake” OR “protein intake” OR “whey concentrate” OR “casein concentrate” OR “concentrate protein” OR “whey isolated” OR “casein isolated” OR “isolated protein” OR “hydrolyzed whey” OR “hydrolyzed casein” OR “hydrolyzed protein” OR milk OR “milk protein” OR soy OR “soy protein”) AND (muscle OR “mass gain” OR “muscle gain” OR “muscular gain” OR “muscle strength” OR “muscular strength” OR “hypertrophy” OR “lean mass” OR “body composition” OR “lean body mass” OR “lean body

tissue" OR "fat-free mass" OR "fat free mass" OR "body weight" OR "body mass" OR "skeletal muscle") NOT (child OR pediatric OR elder OR aged OR rat OR mice)

Search strategy for: Guo Y, Fu X, Hu Q, et al. The Effect of Leucine Supplementation on Sarcopenia-Related Measures in Older Adults: A Systematic Review and Meta-Analysis of 17 Randomized Controlled Trials. Front Nutr. 2022;9:929891. "Supplementary table 1. Literature search strategy for meta-analysis"

#1: Search "L-Leucine"[Mesh] OR "amino acid"[tiab] OR "L-isomer Leucine"[tiab] OR "leucine"[tiab] OR "Leu"[tiab];<br>#2: Search "Sarcopenia";<br>#3: Search "RCT" OR "controlled trial" OR "randomized trial";<br>#4: Search #1 AND #2 AND #3.

Search strategy for: Cheng SH, Chen KH, Chen C, et al. The Optimal Strategy of Vitamin D for Sarcopenia: A Network Meta-Analysis of Randomized Controlled Trials. Nutrients. 2021;13(10):3589. "Supplementary text 1. Search strategy"

**Primary search strategy**<br>#1. sarcopenia<br>#2. sarcopenic<br>#3. #1 OR #2<br>#4. vitamin D<br>#5. vitamin D3<br>#6. ergocalciferol derivative<br>#7. dihydrotachysterol<br>#8. 25 hydroxyvitamin D

#9. colecalciferol derivative

#10. #4 OR #5 OR #6 OR #7 OR #8 OR #9

#11. #3 AND #10

Search strategy for: Wang H, Huang WY, Zhao Y. Efficacy of Exercise on Muscle Function and Physical Performance in Older Adults with Sarcopenia: An Updated Systematic Review and Meta-Analysis. Int J Environ Res Public Health. 2022;19(13):8212. "Supplementary S1. Search strategy"

PubMed (abstract/title/keywords)

(("sarcopenia"[Title/Abstract] OR "sarcopen*"[Title/Abstract]) AND ("exercise"[Title/Abstract] OR "exercis*"[Title/Abstract] OR ("exercis*"[Title/Abstract] AND ("endurance"[Title/Abstract] OR "aerobic"[Title/Abstract] OR "resistance"[Title/Abstract] OR "balance"[Title/Abstract] OR "flexibility"[Title/Abstract] OR "combi*"[Title/Abstract] OR "mixed"[Title/Abstract] OR "multi*"[Title/Abstract] OR "eccentric"[Title/Abstract] OR "eccentric"[Title/Abstract] OR "concentric"[Title/Abstract] OR "isometric"[Title/Abstract] OR ("plyometric"[All Fields] OR "plyometrics"[All Fields]))) OR "walk*"[Title/Abstract] OR "run"[Title/Abstract]) AND ("muscle mass"[Title/Abstract] OR "muscle strength"[Title/Abstract] (("sarcopenia"[Title/Abstract] OR "sarcopen*"[Title/Abstract]) AND ("exercise"[Title/Abstract] OR "exercis*"[Title/Abstract] OR ("exercis*"[Title/Abstract] AND ("endurance"[Title/Abstract] OR "aerobic"[Title/Abstract] OR "resistance"[Title/Abstract] OR "balance"[Title/Abstract] OR "flexibility"[Title/Abstract] OR "combi*"[Title/Abstract] OR "mixed"[Title/Abstract] OR "multi*"[Title/Abstract] OR "eccentric"[Title/Abstract] OR "eccentric"[Title/Abstract] OR "concentric"[Title/Abstract] OR "isometric"[Title/Abstract] OR ("plyometric"[All Fields] OR "plyometrics"[All Fields]))) OR "walk*"[Title/Abstract] OR "run"[Title/Abstract]) AND ("muscle mass"[Title/Abstract] OR "muscle strength"[Title/Abstract] OR "muscle function"[Title/Abstract] OR "fat-free mass"[Title/Abstract] OR "lean mass"[Title/Abstract] OR ("musc*"[Title/Abstract] AND ("function*"[Title/Abstract] OR "power"[Title/Abstract] OR "size"[Title/Abstract] OR "volume"[Title/Abstract] OR "thick*"[Title/Abstract] OR "enlarge*"[Title/Abstract] OR ("frailty"[MeSH Terms] OR "frailty"[All

Fields] OR "weakness"[All Fields] OR "weaknesses"[All Fields]))) OR ("physical"[Title/Abstract] AND ("fitness"[Title/Abstract] OR "function*"[Title/Abstract] OR "capacity"[Title/Abstract] OR "disability"[Title/Abstract] OR ("perform"[All Fields] OR "performable"[All Fields] OR "performance"[All Fields] OR "performance s"[All Fields] OR "performances"[All Fields] OR "performative"[All Fields] OR "performatively"[All Fields] OR "performatives"[All Fields] OR "performativities"[All Fields] OR "performativity"[All Fields] OR "performed"[All Fields] OR "performer"[All Fields] OR "performer s"[All Fields] OR "performers"[All Fields] OR "performing"[All Fields] OR "performs"[All Fields])))) AND ("aged"[Title/Abstract] OR "aging"[Title/Abstract] OR "geriatric*"[Title/Abstract] OR "old"[Title/Abstract] OR "ageing"[Title/Abstract] OR "senior"[Title/Abstract] OR "elder*"[Title/Abstract] OR "adult*"[Title/Abstract])) AND ((fha[Filter]) AND (clinicaltrial[Filter]) AND (humans[Filter]) AND (english[Filter])) OR "muscle function"[Title/Abstract] OR "fat-free mass"[Title/Abstract] OR "lean mass"[Title/Abstract] OR ("musc*"[Title/Abstract] AND ("function*"[Title/Abstract] OR "power"[Title/Abstract] OR "size"[Title/Abstract] OR "volume"[Title/Abstract] OR "thick*"[Title/Abstract] OR "enlarge*"[Title/Abstract] OR ("frailty"[MeSH Terms] OR "frailty"[All Fields] OR "weakness"[All Fields] OR "weaknesses"[All Fields]))) OR ("physical"[Title/Abstract] AND ("fitness"[Title/Abstract] OR "function*"[Title/Abstract] OR "capacity"[Title/Abstract] OR "disability"[Title/Abstract] OR ("perform"[All Fields] OR "performable"[All Fields] OR "performance"[All Fields] OR "performance s"[All Fields] OR "performances"[All Fields] OR "performative"[All Fields] OR "performatively"[All Fields] OR "performatives"[All Fields] OR "performativities"[All Fields] OR "performativity"[All Fields] OR "performed"[All Fields] OR "performer"[All Fields] OR "performer s"[All Fields] OR "performers"[All Fields] OR "performing"[All Fields] OR "performs"[All Fields])))) AND ("aged"[Title/Abstract] OR "aging"[Title/Abstract] OR "geriatric*"[Title/Abstract] OR "old"[Title/Abstract] OR "ageing"[Title/Abstract] OR "senior"[Title/Abstract] OR "elder*"[Title/Abstract] OR "adult*"[Title/Abstract])) AND ((fha[Filter]) AND (clinicaltrial[Filter]) AND (humans[Filter]) AND (english[Filter]))

Selection of eligible studies

Trial registers and databases including PubMed, Cochrane library, Embase, and Scopus were searched for studies published up to December 5, 2022. The results of the database searches were exported to an EndNote 20 library. Duplicates were deleted using the EndNote 20 deduplication function. Two reviewers (MCC and YJC) removed irrelevant records based on the titles and abstracts. Subsequently, the reviewers examined the full text to select articles that meet the selection criteria.

Each database was searched with the following criteria:

1. PubMed (105)
   - Article type: randomized controlled trials
   - Language: English

2. Cochrane library (285)
   - trials

3. Embase (570)
   - Publication type: article
   - Language: English

4. Scopus (4856)
   - Document types: article
   - Publication stage: final
   - Source types: journal
   - Language: English

Table S1. Eligibility criteria

**Population**	Partients diagnosed with sarcopenia.
**Intervention**	Use of whey protein, leucine, and vitamin D supplementation.
**Comparison**	Use of isocaloric supplementation.
**Outcome**	Studies were eligible for inclusion in this review if they report on the muscle mass, muscle strength, or muscle function.
**Study design**	Randomized controlled trials in humans and papers written in English were included in this review.
**Limitation**	Studies published as reviews, letters, or other undistinctive forms were excluded. Studies from all years were considered.
